# Open Surgical Repair for a Giant Common Hepatic Artery Aneurysm with Difficulty in Proximal Arterial Clamping: A Case Report

**DOI:** 10.3400/avd.cr.20-00070

**Published:** 2020-12-25

**Authors:** Naoki Asano, Takashi Yamauchi, Kazunori Ota, Kazuho Niimi, Masahito Saito, Shigeyoshi Gon, Kei Torikai, Shinichi Ban, Hiroshi Takano

**Affiliations:** 1 Department of Cardiovascular Surgery, Dokkyo Medical University Saitama Medical Center, Koshigaya, Saitama, Japan; 2 Department of Pathology, Dokkyo Medical University Saitama Medical Center, Koshigaya, Saitama, Japan

**Keywords:** aneurysm, balloon occlusion, hepatic artery

## Abstract

Hepatic artery aneurysm has been considered as a rare, life-threatening disease. In this study, we report on a patient requiring surgical treatment for a giant hepatic artery aneurysm by aneurysmectomy without revascularization. A 70-year-old woman who complained of epigastric pain was referred to our hospital. Enhanced computed tomography scan has revealed a giant (11×9 cm) common hepatic artery aneurysm. She then underwent emergency surgery; the intra-aortic balloon occlusion technique was applied in order to control the blood inflow into the aneurysm. The aneurysm was then incised, and direct closure of the inflow and outflow orifices was performed safely without evidence of ischemic change in the liver.

## Introduction

Hepatic artery aneurysm (HAA) has been considered to be relatively rare. Although successful endovascular treatment has been reported,^1,2)^ this approach is not feasible for some patients due to anatomical reasons.^3,4)^ In such cases, open surgical repair is performed, and control of the proximal blood flow is necessary. In this study, we present an extremely rare case of a giant common HAA wherein its huge size made the typical proximal clamping site in the abdominal cavity inaccessible. We, therefore, used the intra-aortic balloon occlusion technique to control the proximal blood flow.

## Case Report

**Figure 1:**
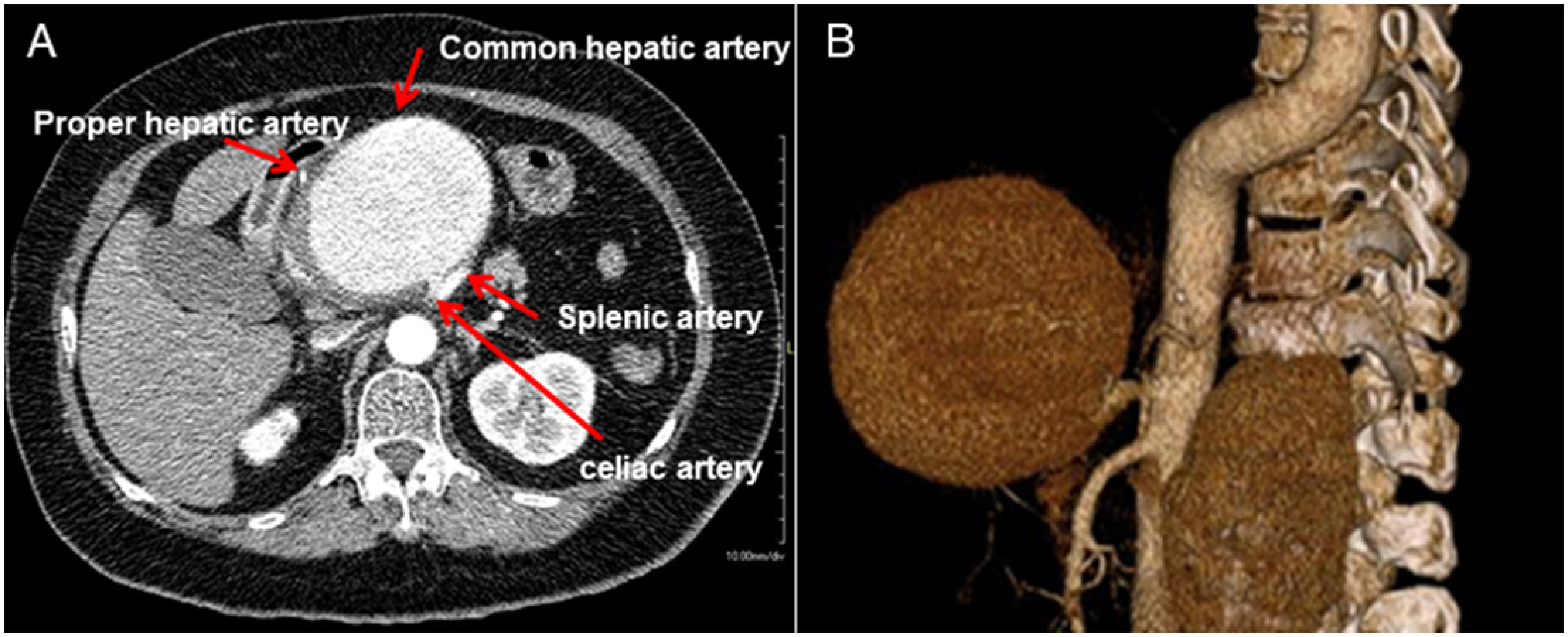
Fig. 1 (**A**) Computed tomography image showing the hepatic artery aneurysm. The size of the aneurysm was 11×9 cm. The orifice of inflow into the aneurysm started 12 mm distal to the origin of the celiac artery after branching of the splenic artery. The proper hepatic artery and gastroduodenal artery ran very close to the aneurysm. (**B**) Three-dimensional computed tomography image of the aneurysm.

A 70-year-old woman with a history of hypertension and diabetes mellitus complained of epigastric pain. During an enhanced computed tomography (CT) scan, a visceral artery aneurysm in the upper abdomen has been revealed. She was immediately referred to our hospital for treatment of aneurysm. Physical examination revealed a pulsatile epigastric mass. CT imaging revealed an 11×9 cm aneurysm, with a substantial mural thrombosis. The orifice of inflow into the aneurysm started 12 mm distal to the origin of the celiac artery, and the splenic artery originated from the celiac artery just proximal to the aneurysm (**Fig. 1A**). Although the proper hepatic artery and the gastroduodenal artery have been determined to run very close to the aneurysm, the location of the outflow orifice was found to be less apparent. The preoperative diagnosis was aneurysm of the common hepatic artery with impending rupture. Emergent open surgical repair was undertaken due to uncertainty regarding the anatomical suitability for endovascular treatment.

**Figure 2:**
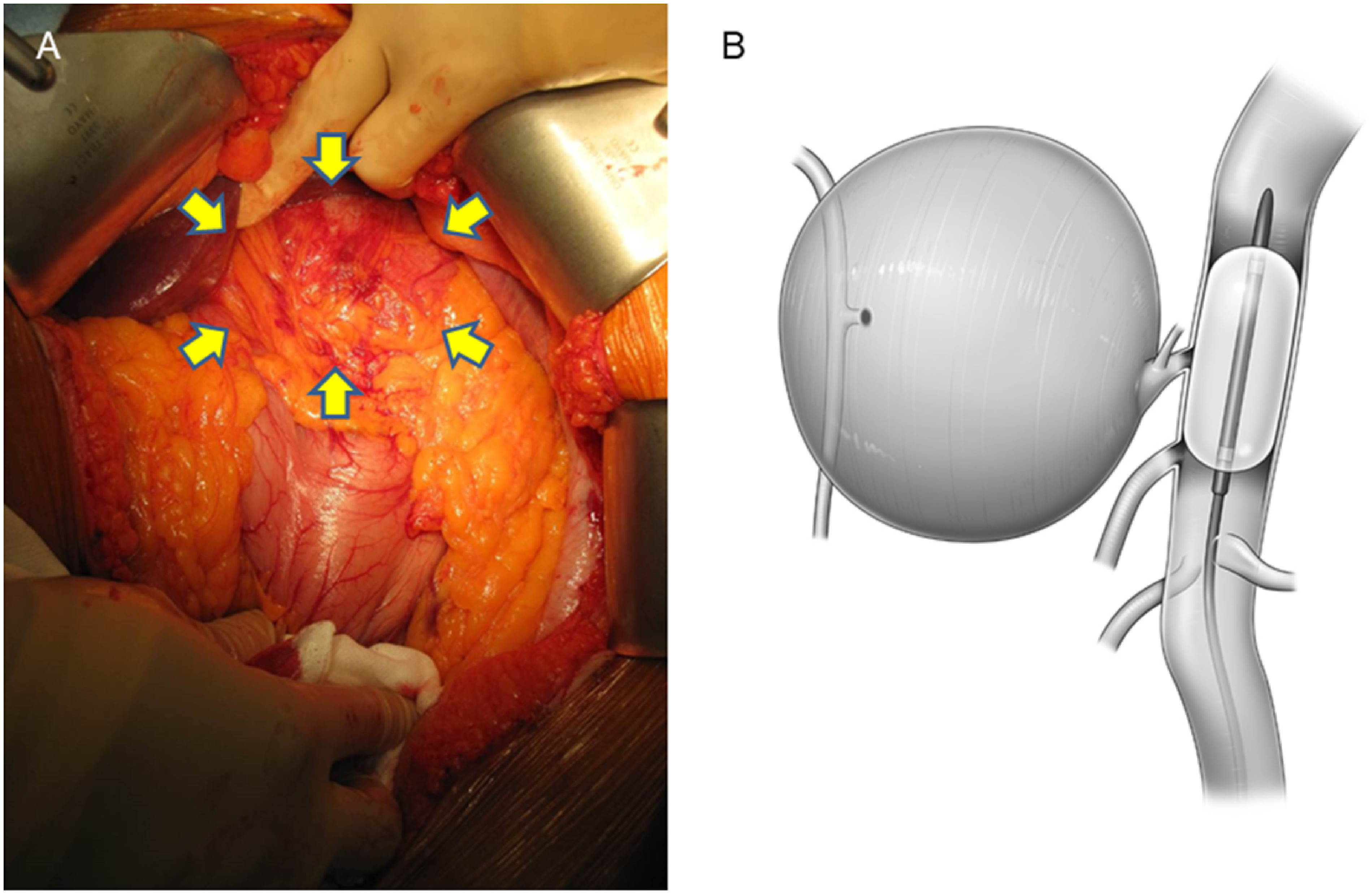
Fig. 2 (**A**) The surface of the aneurysm was confirmed through gastrohepatic omentum. (**B**) The occlusion balloon was then delivered to the orifice of the celiac artery in order to block blood inflow.

Because of the size of the aneurysm, clamping of the proximal artery to block antegrade blood flow seemed difficult (**Fig. 1B**); therefore, intra-aortic balloon occlusion technique was instead utilized. A 14 French (Fr) GORE® DrySeal Flex introducer sheath with a hydrophilic coating (W. L. Gore & Associates, Inc., Flagstaff, AZ, USA) was then inserted into the left femoral artery, and a 12 Fr Reliant stent graft balloon catheter for occlusion (Medtronic Vascular, Inc., Santa Rosa, CA, USA) was placed in the abdominal aorta at the level of the celiac artery. After the upper midline laparotomy, the surface of the aneurysm was confirmed through gastrohepatic omentum (**Fig. 2A**). The occlusion balloon was then inflated, and disappearance of pulsation of the aneurysm was confirmed (**Fig. 2B**). We did not perform an occlusion test of the arterial supply to the liver because it has been shown that vascular occlusions of the liver can be extended safely for up to 60 min.^5)^ Gross examination of the aneurysm showed no inflammatory or posttraumatic changes such as wall thickening or dense adhesion to the surrounding tissue. Then, the aneurysm was incised, and mural thrombus was removed. The inflow orifice was located on the dorsal side of the aneurysm, while the outflow orifice was located on the right side. Some bleeding was observed from the outflow orifice of the aneurysm, which was taken care of by finger compression of the orifice.

Direct closure of the inflow and outflow orifices was easily performed from the inside. The balloon occlusion time was determined to be around 9 min. The continuity of the proper hepatic artery and gastroduodenal artery was confirmed visually. After visual inspection to ensure that the liver showed no ischemic change, the laparotomy was closed. Intraoperative blood loss was determined to be at 450 mL.

The postoperative course was determined to be uneventful except for a slight elevation in hepatic enzymes with maximum aspartate transaminase 121 IU/L (reference range 13–30 IU/L) and maximum alanine transaminase 110 IU/L (reference range 7–23 IU/L). The patient was discharged home on postoperative day 12 and continued to be well throughout the 3-year follow-up after surgery.

**Figure 3:**
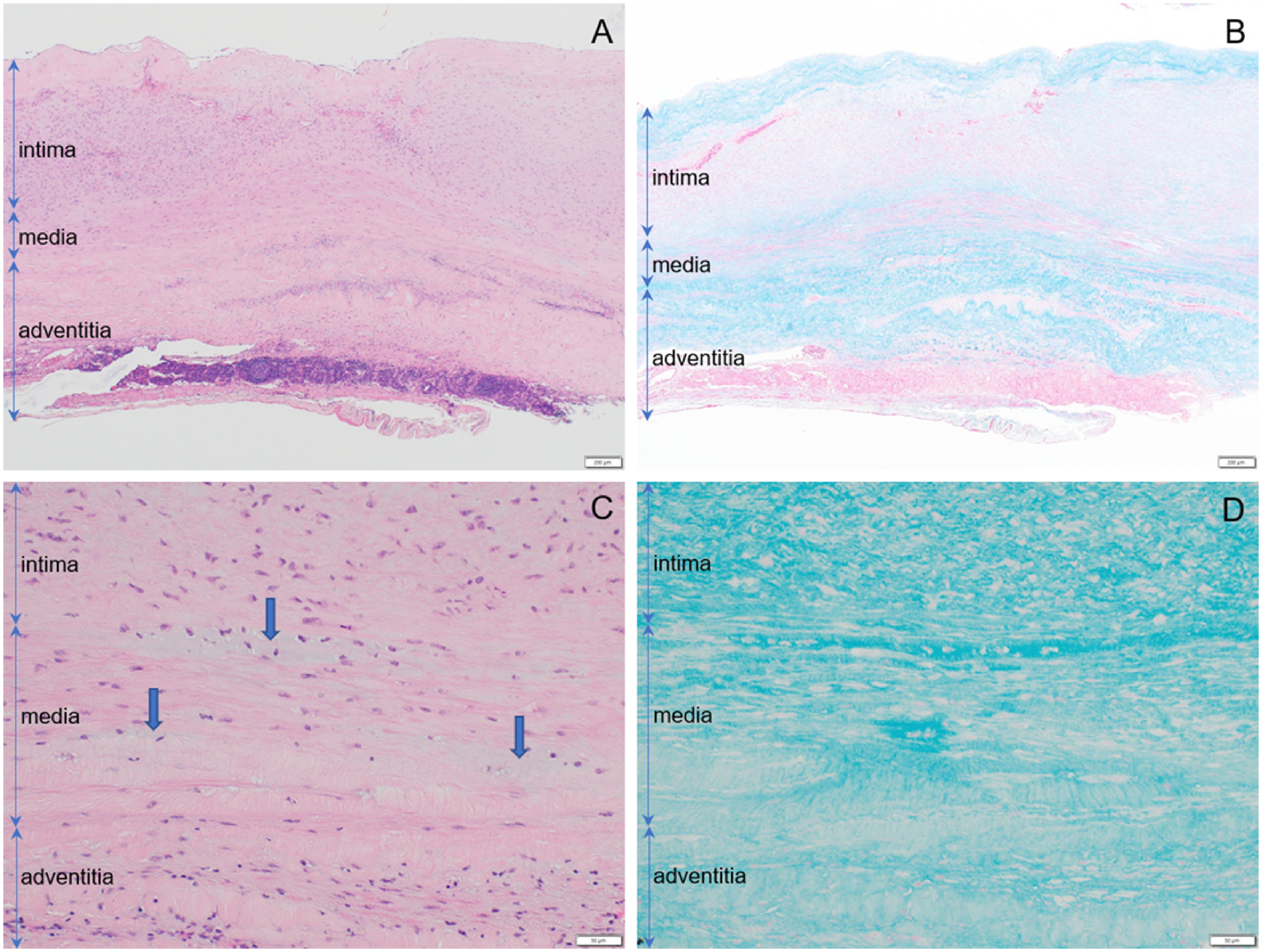
Fig. 3 (**A**, **B**) Histopathological examination of cross sections of the aneurysm wall revealed thickening of the intima with fibromyxoid hyperplasia, dense fibrosis of the media and adventitia with neovascularization and focal residual thin layers of smooth muscle cells of the media in the subintima, and focal lymphoplasmacytic infiltration in the adventitia (**A**: hematoxylin–eosin stain, **B**: Azan-Mallory stain, scale bar 200 µm). (**C**, **D**) High-power images of the aneurysm wall with myxoid change of the residual thin smooth muscle layers (**C**, arrows), and the deposition of a mucosubstance in all layers of the aneurysm wall (**D**). (**C**: hematoxylin–eosin stain, **D**: Alcian blue stain, scale bar 50 µm).

Histopathological examination of cross sections of the aneurysm wall has revealed thickening of the intima with fibromyxoid hyperplasia, and dense fibrosis of the media and adventitia with neovascularization and focal residual thin layers of smooth muscle cells of the media in the subintima (**Figs. 3A** and **3B**). The internal elastic lamina had mostly disappeared with a few residual fragments. Myxoid change has been observed in the residual thin smooth muscle cell layers (**Fig. 3C**), and Alcian blue staining was able to reveal the deposition of a mucosubstance in all layers of the aneurysm wall (**Fig. 3D**). Typical atheromatous change was not obvious. Focal lymphoplasmacytic infiltration was then observed in the adventitia, and CD138 immunohistochemistry confirmed the presence of plasma cells; however, these cells were found to be not immunoreactive for IgG4. No active inflammation with fibrinoid necrosis or granulomatous change with any multinucleated giant cells was observed. Consequently, atherosclerosis was not identified to be the main cause of aneurysm; furthermore, associating aneurysm with IgG4-related disease or systemic vasculitis syndrome was deemed not plausible. The pathologic findings were not consistent with fibromuscular dysplasia. Association with infection, trauma, drugs, and any collagen diseases or Kawasaki disease was also not supported clinically. Thus, considering the myxoid degeneration of the residual medial smooth muscle layers and the deposition of a mucosubstance in all layers of the aneurysm wall, any degenerative abnormality of the media that impaired the elasticity of the arterial wall would be the likely etiology for the development of the aneurysm.

## Discussion

HAA has been identified as a rare disease that carries a high risk of death if left untreated. Among the visceral aneurysms, the incidence of HAA is estimated to be around 20%.^6)^ About 80% of HAAs are extrahepatic, and the common hepatic artery is the most frequent site.^7)^ The most typical symptoms are epigastric pain, gastrointestinal hemorrhage, and jaundice (Quincke’s triad). However, most patients with HAA are asymptomatic, and approximately 80% of these patients are not diagnosed until the aneurysm ruptures. Once HAA is found, prompt repair should be conducted as mortality increases up to 40% after rupture.^8)^

Treatment options for HAA include either open surgical repair or endovascular intervention. Although open repair is a conventional and reliable method of treatment, reports on endovascular treatment of HAA are also increasing. To the best of our knowledge, at least 10 cases of endovascular treatment for common HAA have been reported in Japan. The best treatment modality for each case is still controversial, and it still further depends on clinical presentation, type and location of the aneurysm, associated risk factors, and general status of the patient.^9)^ In general, intrahepatic aneurysm, saccular aneurysm with a narrow neck, and downstream organ fed by another (nonaneurysmal) artery are considered favorable conditions for endovascular treatment.^8)^ Large size itself is not a contraindication for endovascular treatment, and favorable results have been reported in patients with giant HAA.^1,2)^ In this study, we opted for an open surgical repair in this patient as the splenic artery originated just proximal to the aneurysm, and embolization of the celiac axis can occlude the splenic artery. Additionally, there was insufficient information regarding the outflow orifice of the aneurysm.

When common HAA is excised in open surgery, it is essential to confirm the continuity of the superior mesenteric artery, pancreaticoduodenal artery, gastroduodenal artery, and proper hepatic artery=to be able to judge the necessity of revascularization. If the continuity of those arteries and collateral circulation to the liver are confirmed, simple resection of the HAA without revascularization can be performed. However, Imazuru et al.^10)^ reported that the possibility of postoperative cholecystitis after HAA resection without revascularization should be considered. Fortunately, this complication did not develop in our patient.

Proximal and distal blood flow blockage is desirable for safe aneurysmal resection. As the huge size of the aneurysm in this patient has prevented the clamping of the proximal artery, we utilized the intra-aortic balloon occlusion technique as reported by Angiletta et al.^4)^ Selective occlusion of the celiac trunk may be less invasive compared with aortic occlusion. However, we used the intra-aortic balloon occlusion technique because it is easier and faster than the selective celiac occlusion technique, and it can decrease bleeding not only from the inflow (through the celiac trunk) but also from the outflow orifices (through the pancreaticoduodenal arcade and gastroduodenal artery). Additionally, because the proximal neck of the aneurysm was short, the selective occlusion balloon catheter might end up disturbing the suturing of the inflow orifice of the aneurysm, and thus dissection and clamping of the celiac trunk will be necessary after opening the aneurysm.

## Conclusion

Open surgical treatment has been identified as an effective procedure in treating a giant HAA. If the proximal clamping site is inaccessible because of the large size of the aneurysm, intra-aortic balloon occlusion technique is preferred as it is feasible and useful for treatment.
